# *In Vitro* Leishmanicidal Effect of Silibinin: Disrupting Redox Balance via Trypanothione Reductase Inhibition

**DOI:** 10.1021/acsomega.5c02606

**Published:** 2025-06-18

**Authors:** Natalia Debize da Motta, Luiza Gervazoni Ferreira de Oliveira, Myslene Soares da Fonseca, Job Domingos Inacio Filho, Elmo Eduardo de Almeida-Amaral

**Affiliations:** Laboratório de Bioquímica de Tripanosomatídeos, 37903 Instituto Oswaldo Cruz/Fundação Oswaldo Cruz, Rio de Janeiro 21040-360, Brazil

## Abstract

Visceral leishmaniasis, caused by the parasite *Leishmania infantum*, is a life-threatening disease with limited therapeutic options that are often associated with toxicity and resistance. In this study, we investigated the *in vitro* leishmanicidal effects of silibinin, a key flavonolignan from *Silybum marianum*, against both the promastigote and intracellular amastigote forms of *L. infantum*. Mechanistically, silibinin inhibits trypanothione reductase (TR), disrupting the redox balance in the parasite and causing cell death. Silibinin concentration-dependently inhibited promastigote proliferation (IC_50_ of 416.7 μM) with potent activity against intracellular amastigotes (EC_50_ of 0.7 μM) and a high selectivity index (242), indicating its strong therapeutic potential and nontoxicity to macrophages. Importantly, silibinin disrupts the *L. infantum* redox balance by inhibiting TR activity, which increases the reactive oxygen species (ROS) levels to kill the parasite. ROS accumulation and the inhibition of parasite proliferation were significantly correlated (Pearson correlation coefficient of 0.9895). Molecular docking confirmed that silibinin binds to the catalytic site of TR, corroborating its role in ROS-mediated parasite death. Furthermore, *in silico* ADMET analysis revealed that silibinin has favorable pharmacokinetic properties for oral administration. Finally, *in vitro* and *in silico* studies indicated that silibinin inhibits TR, a key enzyme in *Leishmania* redox homeostasis, to exert antileishmanial effects.

## Graphical abstract

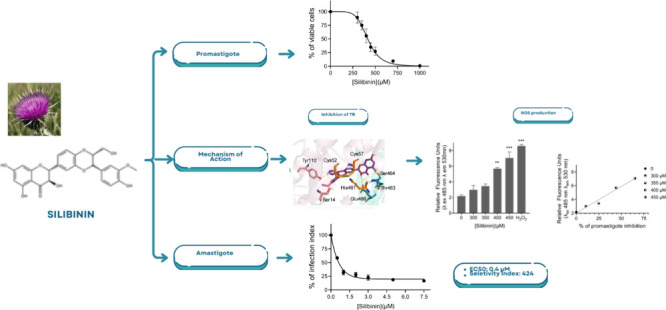

## Introduction

Visceral leishmaniasis, caused by the protozoan parasite *Leishmania infantum*, presents a significant global public health concern. It is known to be a potentially fatal disease, ranking second and seventh among tropical diseases in terms of mortality and loss of disability-adjusted life years.^1^

This neglected tropical disease predominantly affects vulnerable populations in developing countries, with an estimated 50,000 to 90,000 new cases reported annually, 90% of which are reported in seven countries (Brazil, Ethiopia, India, Kenya, Somalia, South Sudan, and Sudan).^2^ If left untreated, the clinical manifestations of visceral leishmaniasis can be severe, leading to mortality in up to 95% of cases.^3^

The current options for treating leishmaniasis mainly consist of pentavalent antimonials, which have been developed over many years and are typically given in hospital environments. However, the efficacy of these drugs has decreased due to the increase in drug resistance. Amphotericin B has emerged as an alternative leishmaniasis therapy, and its formulation into liposomes has been intended to significantly lessen the dose-related negative side effects and shorten the long treatment duration. However, this formulation is associated with high costs.^2^

Paromomycin has been approved for leishmaniasis treatment in India, but its effectiveness in Africa remains to be confirmed. One of the most promising treatments that has emerged in recent years is miltefosine, which is the first oral compound authorized for leishmaniasis therapy. Miltefosine is available in India and several other nations and recently gained approval from the FDA under the trade name Impavido for the treatment of both visceral and cutaneous leishmaniasis. Although miltefosine is effective, it is also expensive and has teratogenic properties.^4^

Natural products have been recognized in pharmacology for their therapeutic potential, a tradition that traces back to ancient civilizations. Natural products have also played crucial roles in the discovery and development of new drugs, often via the modification of their chemical structures. In recent years, there has been considerable interest in natural products, especially flavonoids, as promising sources of new therapeutic agents for a range of diseases, including parasitic infections.^5^

Among these flavonoids, flavonolignans derived from the seeds of *Silybum marianum* (L.) Gaernt (Sm.), commonly known as the milk thistle, have emerged as compelling candidates for further investigation in the context of visceral leishmaniasis treatment.^6^

Silibinin is the main flavonolignan present in *S. marianum* (L.) Gaernt (Sm.) extract (silymarin). In nature, silibinin occurs in the form of two diastereoisomers in an approximately quasi-equimolar ratio.^7^

The main feature of silibinin is its hepatoprotective properties.^8^ Nevertheless, other pharmacological activities of this compound have been extensively investigated, including antioxidant, anti-inflammatory, anticancer, and antiparasitic effects^6,8–10^ Previous research has also demonstrated its potential as a multitarget therapeutic agent because of its ability to modulate various cellular pathways.^8,11^

This study aimed to assess the efficacy of silibinin against *L. infantum* and elucidate its mechanism of action, thereby contributing to the development of innovative treatment strategies for visceral leishmaniasis. These findings could help address unmet medical needs and advance research toward the goals of disease control and elimination.

## Results and Discussion

### Leishmanicidal Activity of Silibinin against the Promastigote and Intracellular Amastigote Forms of *Leishmania infantum*

To investigate the leishmanicidal effect of silibinin, we first analyzed its effects on the proliferation of *L. infantum* promastigotes exposed to various concentrations (300–1000 μM) for 72 h. The results indicated concentration-dependent inhibition of cellular proliferation with an IC_50_ of 416.7 μM (Figure 1). Notably, at a concentration of 1000 μM, silibinin completely inhibited the proliferation of *L. infantum* after 72 h.

**Figure 1:**
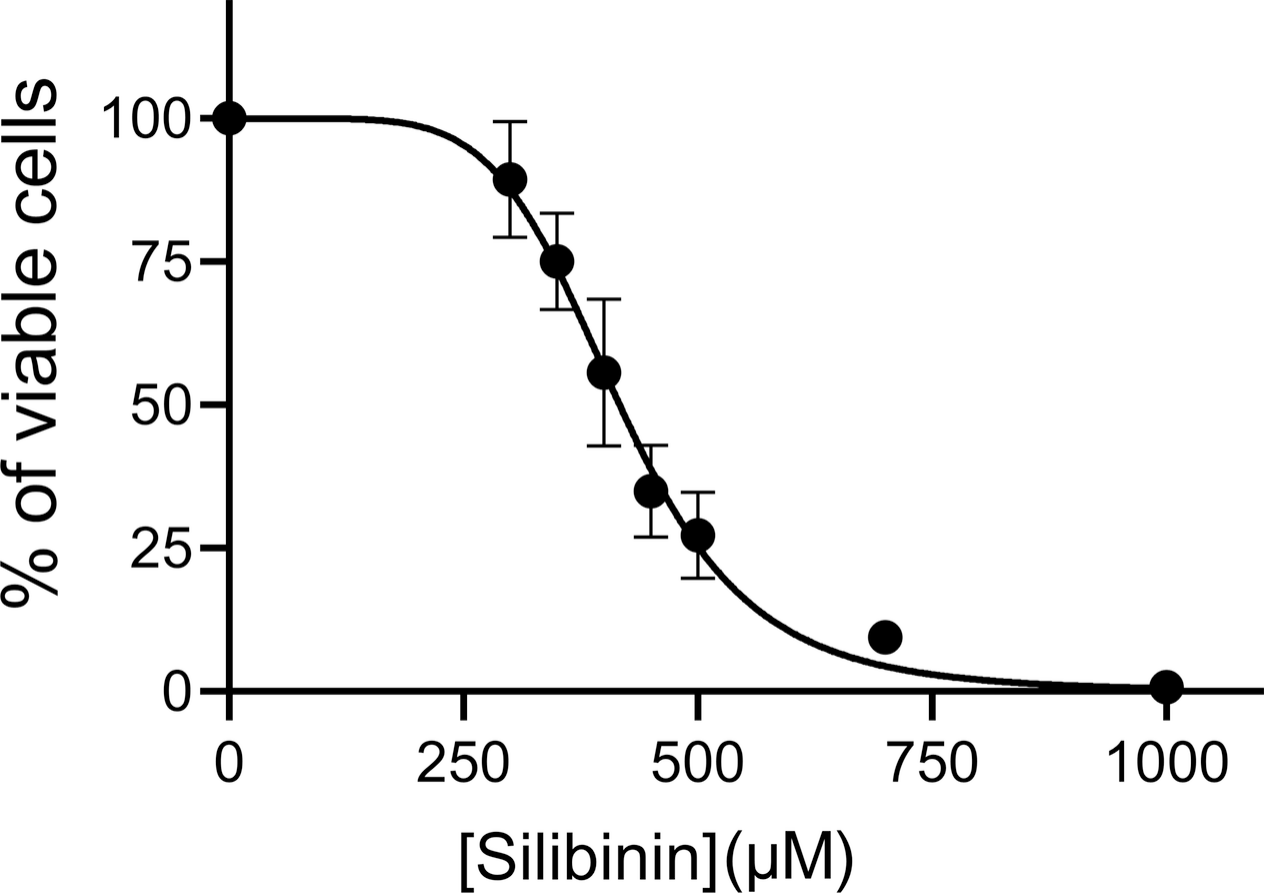
Viability of *Leishmania infantum* promastigotes treated with silibinin. *L. infantum* promastigotes were cultured in Schneider’s *Drosophila* medium at 26 °C for 72 h in the absence or presence of silibinin (300, 350, 400, 450, 500, 700, or 1000 μM). Cell viability was assessed using the Alamar Blue assay. In the control group (absence of silibinin), 0.2% DMSO (vehicle) was added to the growth medium. Data represent the mean percentage ± standard error of three independent experiments. ANOVA, *p* < 0.001.

To analyze the effects of silibinin on *L. infantum* intracellular amastigotes, peritoneal BALB/c mouse macrophages were infected with the promastigotes of *L. infantum* for 4 h. Following this, they were exposed to increasing concentrations of silibinin ranging from 0.5 to 7.5 μM, for 72 h.

Silibinin reduced the infection index in a concentration-dependent manner (Figure 2a), exhibiting an EC_50_ of 0.7 μM and achieving 97.7% inhibition at the highest tested concentration (7.5 μM). Additionally, an assessment of the cytotoxic effect of silibinin indicated that it was nontoxic (Figure 2b). Logarithmic regression (*R* ^2^ = 0.93) of this concentration-dependent curve revealed a CC_50_ value of 169.6 μM, resulting in a selectivity index of 242. Notably, a drug’s biological efficacy is not linked to cytotoxicity when the selectivity index is greater than or equal to 10.^12^ Furthermore, the antileishmanial activity of silibinin against *L. infantum*-infected macrophages was superior to those of miltefosine and other flavonoids.^13^

**Figure 2:**
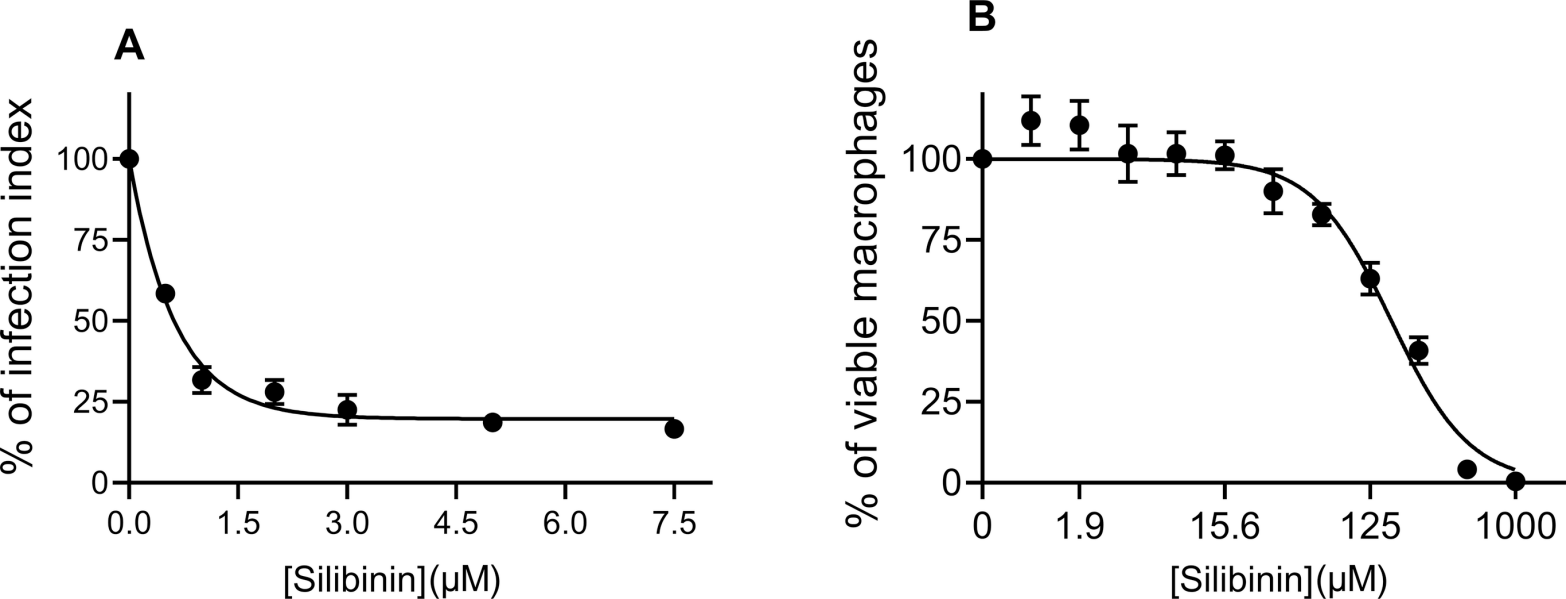
Antiamastigote efficacy of silibinin and its toxicity to murine macrophages. (A) Peritoneal murine macrophages were infected (MOI 5:1) with stationary-phase *L. infantum* promastigotes for 4 h at 37 °C with 5% CO_2_ and then further incubated in the absence or presence of various concentrations of silibinin (0.5, 1, 2, 3, 5, or 7.5 μM) for 72 h. The infection index was determined via light microscopy by counting at least 200 macrophages per coverslip in duplicate. The results are expressed as a percentage of infection index (% of infected macrophages × number of amastigotes/total number of macrophages). (B) Peritoneal murine macrophages were plated and treated with different concentrations of silibinin (0.98–1000 μM). Data represent the mean ± standard error of three independent experiments. ANOVA, *p* < 0.001. The 100% value refers to 307.3 ± 51.5. In the control group (absence of silibinin), 0.2% DMSO (vehicle) was added to the growth medium.

The results presented here are in sharp contrast to those previously reported for *Leishmania donovani* ^6^ and *Leishmania major*.^10^ In *L. donovani*, 120 μM silibinin resulted in only a 37% decrease in promastigote proliferation after 72 h, while in *L. major*, 100 μM silibinin led to 77% parasite death over the same period. In our study, complete inhibition of *L. infantum* promastigote proliferation was observed only at 1000 μM silibinin, a concentration approximately 10 times higher than that used in these previous studies. However, the EC_50_ of silibinin against *L. infantum* intracellular amastigotes was remarkably low (0.7 μM), 94.5-fold lower than that observed in *L. major* (66.12 μM). Interestingly, Olias-Molero et al.^6^ noted that 10 μM silibinin did not inhibit *L. infantum* intracellular amastigotes after 24 h of treatment.

These discrepancies may be attributable to the varying susceptibilities of different *Leishmania* species or the study methodology used. Notably, our study employed *L. infantum* (MHOM/MA/67/ITMAP263) and used high-purity silibinin (≥98% by HPLC), whereas Olias-Molero et al.^6^ treated *L. infantum* and *L. donovani* promastigotes with a panel of flavonolignans applied at lower concentrations and for shorter durations and observed no effect against intracellular amastigotes. In contrast, Faridnia et al.^10^ treated *L. major* with silymarin and silibinin at significantly lower concentrations (≤100 μM) and under different culture conditions (RPMI 1640 at 24 °C) and demonstrated only modest efficacy. Furthermore, the purity of silibinin is particularly important because this is considered the primary source of the uncertainty of the results obtained with these compounds that make the biological studies complex and incomparable.^14^

The observed discrepancy between the IC_5__0_ and EC_50_ values for promastigotes and intracellular amastigotes, respectively (Table 1), may be attributable to differences in the developmental stage of the parasite, which can influence the efficacy of the inhibitors. For example, Santos et al.^15^ demonstrated that *Leishmania amazonensis* amastigotes residing within macrophages exhibit greater sensitivity to HIV aspartyl peptidase inhibitors than promastigotes cultured *in vitro* do. This stage-dependent difference in susceptibility may explain the reduced responsiveness of promastigotes to silibinin compared to the heightened sensitivity observed with intracellular amastigotes.

**Table 1:** IC_50_ Values against *L. infantum* Promastigotes, EC_50_ against *L. infantum* Intracellular Amastigotes, CC_50_ against Peritoneal macrophages, and Selectivity Index for Silibinin^a^

promastigotes	intracellular amastigotes	peritoneal macrophages	selectivity index
416.7 ± 62.5 μM	0.7 ± 0.07 μM	169.6 ± 1.1 μM	242

a The IC_50_, EC_50_, and CC_50_ values were determined via logarithmic regression analysis with GraphPad Prism 6 (GraphPad Software, La Jolla, California, USA). The selectivity index was calculated as the macrophage CC_50_/intracellular amastigote EC_50_ ratio.

An additional explanation for the increased efficacy of silibinin against intracellular amastigotes could involve the accumulation of this compound within macrophages. Such accumulation may lead to elevated local concentrations of silibinin at the site of infection, thereby enhancing its antiparasitic activity. For example, studies on *L. infantum* have shown that considerably lower concentrations of HIV-1 protease inhibitors are needed to achieve significant effects against intracellular amastigotes than against axenic amastigotes.^16^

### Theoretical Predictions of the Pharmacokinetic Properties and Oral Bioavailability of Silibinin

To perform *in silico* analysis and evaluate the potential of silibinin as a drug for the treatment of leishmaniasis via the oral route, we used the pkCSM platform^17^ to assess the predicted pharmacokinetic properties (absorption, distribution, metabolism, excretion, and toxicity (ADMET)) of silibinin. Interpretation of the results obtained from the pkCSM database revealed that silibinin exhibited a high probability of human intestinal absorption, appearing to permeate Caco-2 cells, while not being a P-glycoprotein substrate. In terms of metabolism, silibinin is not a CYP substrate but is an inhibitor of CYP2C9, CYP2C19, and CYP1A2. In the toxicity analysis, silibinin exhibited a high probability of lacking toxicity or carcinogenicity (Table 2). We also evaluated its chemical characteristics according to Lipinski’s rule of five.^18,19^ The compound fully satisfied Lipinski’s rule of five, not violating any of the rules (Table 2).

**Table 2:** In Silico ADMET and Lipinski’s Rule of Five Prediction of Silibinin^a^

**category**	**property**	**predicted value**
absorption	water solubility	0.63mmol/L
	Caco-2 permeability	low permeability (2.72 × 10^–6^ cm/s)
	intestinal absorption (human)	61.86%
	P-glycoprotein substrate	yes
	P-glycoprotein I inhibitor	yes
	P-glycoprotein II inhibitor	yes
distribution	volume of distribution (human)	2.34L/kg
	fraction unbound (human)	0
	BBB permeability	poorly distributed (0.062)
	CNS permeability	unable to penetrate (0.00023)
metabolism	CYP2D6 substrate	no
	CYP3A4 substrate	no
	CYP1A2 inhibitor	no
	CYP2C19 inhibitor	no
	CYP2C9 inhibitor	yes
	CYP2D6 inhibitor	no
	CYP3A4 inhibitor	no
excretion	total clearance	0.79mL/min/kg
	renal OCT2 substrate	no
toxicity	AMES toxicity	no
	max. tolerated dose (human)	low (4.47 mg/kg/day)
	hERG I inhibitor	no
	hERG II inhibitor	yes
	oral rat acute toxicity (LD_50_)	2.56mol/kg
	oral rat chronic toxicity (LOAEL)	3.12 mg/kg/day
	hepatotoxicity	no
	skin sensitization	no
Lipinski's rule of five		
molecular weight (≤500)	482.44	0 violations
log P (≤5)	2.363	
HBA (≤10)	10	
HBD (≤5)	5	

a BBB: blood–brain barrier; renal OCT2: renal organic cation transporter 2; hERG I: human ether-a-go-go gene I; hERG II: human ether-a-go-go gene I HIA: human intestinal absorption; log P: logarithm of compound partition coefficient between *n*-octanol and water; HBA: number of hydrogen bond acceptors; HBD: number of hydrogen bond donors.

Bioavailability radar analyses of the physicochemical properties of silibinin using the SwissADME platform also suggested its potential for oral absorption. The pink area of the bioavailability radar indicated a suitable physicochemical space for oral bioavailability, and only a slight infraction in the polarity parameter was observed (Figure 3). These data suggest that this compound is safe and can be orally absorbed.

**Figure 3:**
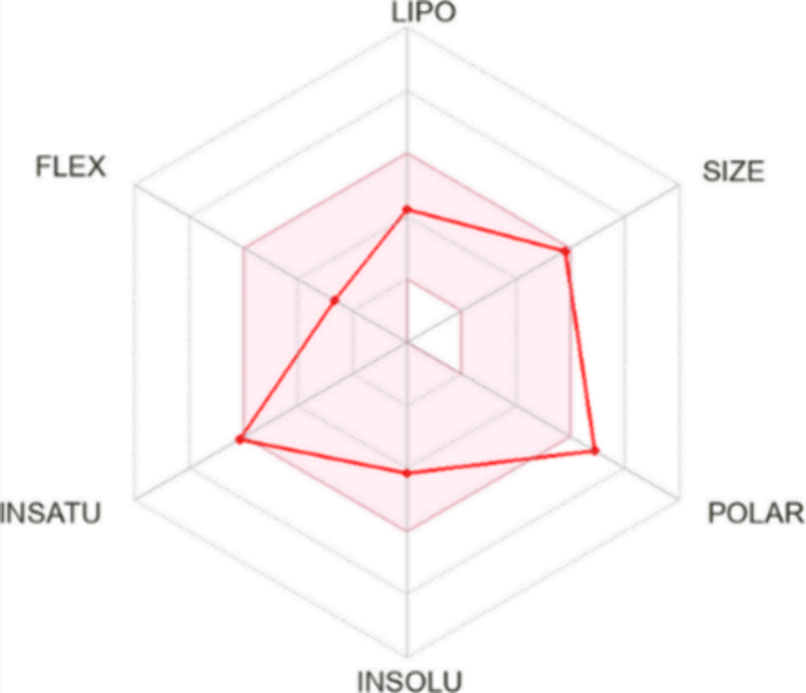
Bioavailability radar analysis of silibinin based on its physicochemical properties. The pink area represents the ideal physicochemical space for oral bioavailability. LIPO, lipophilicity; POLAR, polarity; INSOLU, insolubility; INSATY, unsaturation; and FLEX, flexibility.

### Silibinin Promotes the Inhibition of the Proliferation of *L. infantum* Promastigote by Increasing Intracellular ROS Levels

Flavonoids are known to increase the concentration of reactive oxygen species (ROS) in *Leishmania*.^5,20,21^ To investigate whether this phenomenon also occurs in promastigotes of *L. infantum*, we monitored the levels of ROS in parasites treated with silibinin at concentrations ranging from 300 to 450 μM for 72 h. The ROS levels increased with increasing doses of silibinin. Specifically, the ROS concentration increased 5.1-fold in promastigotes treated with 450 μM silibinin compared to that in nontreated controls (Figure 4a).

**Figure 4:**
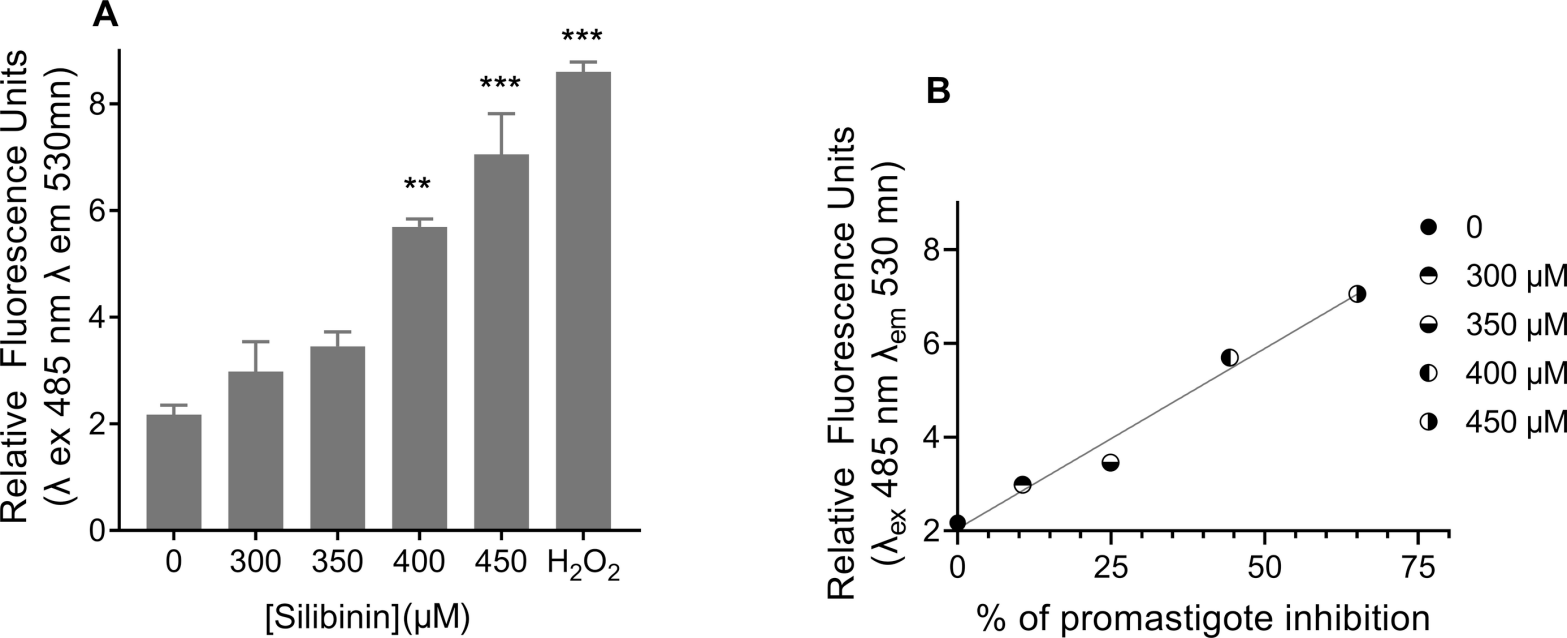
Induction of reactive oxygen species (ROS) in *Leishmania infantum* promastigotes by silibinin. (A) *L. infantum* promastigotes were cultured in Schneider’s *Drosophila* medium at 26 °C for 72 h in the absence or presence of silibinin (300, 350, 400, and 450 μM). ROS production was measured as described in the Experimental Section. Data are expressed as relative fluorescence units (λ excitation 485 nm, λ emission 530 nm). H_2_O_2_ (1 mM) was used as a positive control. Data represent the mean ± standard error of three independent experiments. ** indicates significant difference relative to the control group (*p* < 0.01); *** indicates significant difference relative to the control group (*p* < 0.001). (B) Linear correlation between *L. infantum* promastigote inhibition (*x*-axis) and ROS accumulation (*y*-axis) upon treatment with silibinin. Simple linear correlation (*R* ^2^ = 0.9791); Pearson correlation (*r*) = 0.9895.

There appeared to be a positive correlation between the inhibition of *L. infantum* promastigotes and the concentration of ROS, with a Pearson correlation coefficient of −0.9025 (Figure 4b). This suggests that treatment with silibinin leads to an accumulation of ROS in *L. infantum* promastigotes, directly contributing to parasite death. A similar correlation was noted in promastigotes of *L. infantum* and *Leishmania braziliensis* subjected to relatively high concentrations of EGCG.^20,22^ Such a correlation was also observed in promastigotes and intracellular amastigotes of *L. amazonensis* treated with apigenin, as well as in *L. amazonensis* promastigotes treated with quercetin.^23–25^

### The Increased Intracellular ROS Levels Promoted by Silibinin Are Due to the Inhibition of Trypanothione Reductase (TR) Activity

In *Leishmania* spp. and related trypanosomatid organisms, the redox balance is primarily maintained by trypanothione reductase (TR), a FAD-dependent disulfide oxidoreductase that facilitates the reduction of trypanothione [N1,N8-bis-glutathionylspermidine or T­(SH)_2_] at the expense of NADPH to return trypanothione to its reduced (active) form. This process is crucial for maintaining the redox balance in and overall cell viability of parasites, highlighting the essential nature of TR.^26–28^ Inhibiting TR activity increases H_2_O_2_ levels in the parasite, leading to oxidative stress and the destruction of cellular macromolecules, which promotes parasite death.^20^

Given the observed accumulation of ROS in *L. infantum* promastigotes following incubation with varying concentrations of silibinin, docking studies were conducted to further elucidate the role of TR inhibition in the potential mechanism of action. This enzyme plays an essential role in thiol and redox metabolism in trypanosomatid parasites, and inhibition of its activity leads to ROS accumulation and subsequent parasite cell death.^20,29,30^

Two diastereoisomers of silibinin occur in nature. Therefore, both isoforms, silibinin A and silibinin B (Figure 5), were used in docking calculations for two *L. infantum* TR crystal structures: one in an oxidized state (oxidized TR; PDB code 2JK6) and the other in a reduced state (reduced TR; PDB code 4ADW). Tables 3 and 4 present the results of the interaction calculations for the enzyme-ligand complex. Each of the two ligands exhibited a low Δ*G* value and *K*_i_ in the micromolar range. Isoform silibinin A exhibited binding affinities (Δ*G*) of −6.67 and −5.42 kcal/mol and *K*_i_ values of 12.88 and 106.41 μM for oxidized TR and reduced TR, respectively (Table 3). Additionally, silibinin B exhibited Δ*G* values of −7.18 and −5.25 kcal/mol and *K*_i_ values of 5.61 and 142.59 μM for oxidized TR and reduced TR, respectively (Table 4).

**Figure 5:**
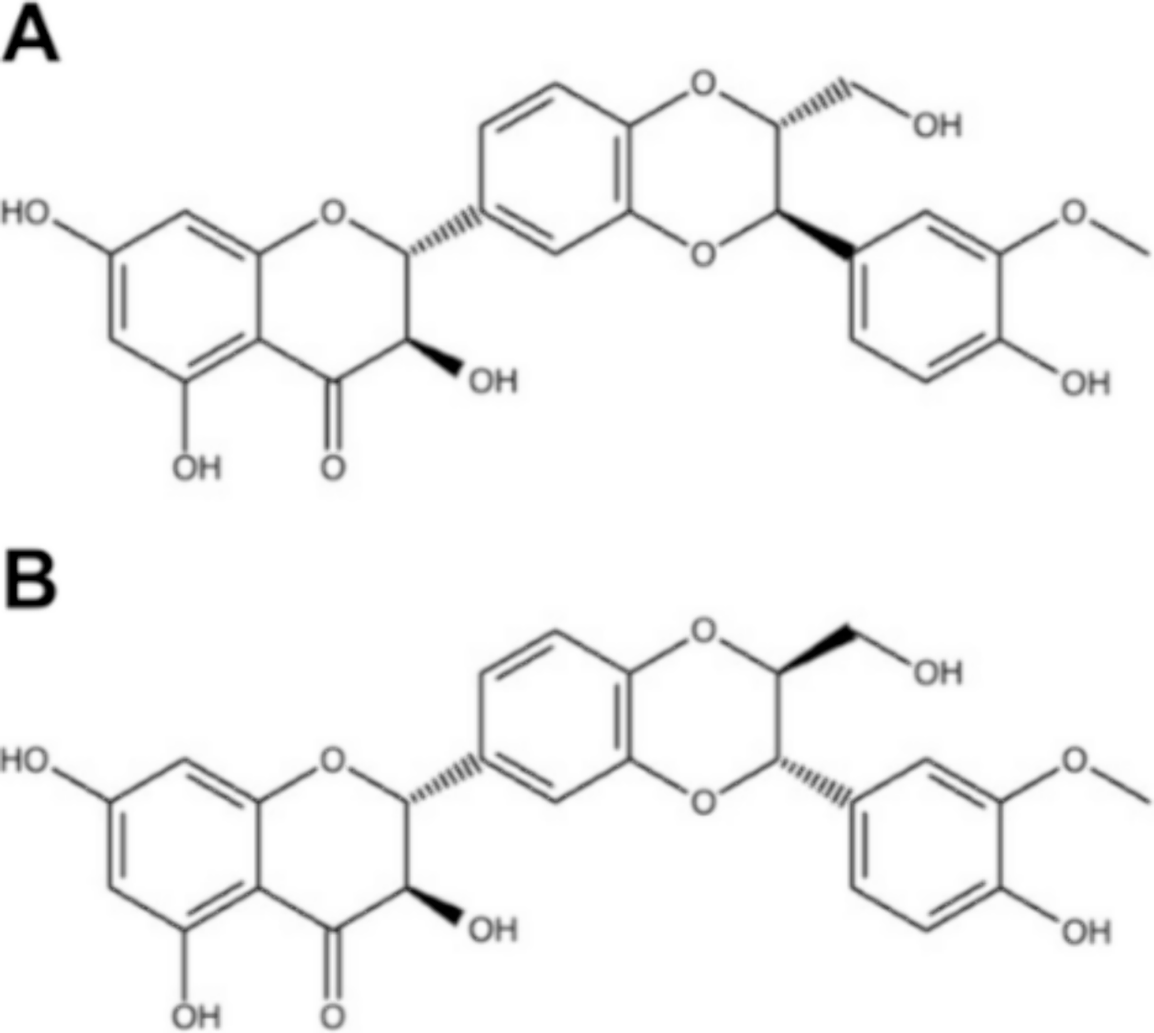
Structural representations of the diastereoisomers of silibinin. (A) Structure of diastereoisomer A. (B) Structure of diastereoisomer B.

**Table 3:** Docking Calculations of Interactions of Silibinin A with the Oxidized (TR^ox^) and Reduced (TR^red^) States of Trypanothione Reductase^a^

state	parameter	value (TR^ox^-2JK6)	value (TR^red^-4ADW)
lower-energy conformation	binding affinity (kcal/mol)	–6.67	–5.42
	inhibition constant (*K*_i_, μM)	12.88	106.41
	rank (cluster/total)	1/59	1/9
	number of poses per cluster	117	127

most prevalent conformation	binding affinity (kcal/mol)	–6.67	–4.95
	inhibition constant (*K*_i_, μM)	12.88	236.53
	rank (cluster/total)	1/59	6/71
	number of poses per cluster	117	127

a Docking simulations were performed using the AutoDock-based Lamarckian genetic algorithm (LGA) with 500 docking runs. The grid map was centered around the active site residues of trypanothione reductase (TR) in both oxidized (TR^ox^, PDB: 2JK6) and reduced (TR^red^, PDB: 4ADW) states.

**Table 4:** Docking Calculations of Interactions of Silibinin B with the Oxidized (TR^ox^) and Reduced (TR^red^) States of Trypanothione Reductase^a^

state	parameter	value (TR^ox^-2JK6)	value (TR^red^-4ADW)
lower-energy conformation	binding affinity (kcal/mol)	–7.18	–5.25
	inhibition constant (*K*_i_, μM)	5.61	142.59
	rank (cluster/total)	1/27	1/21
	number of poses per cluster	109	123

most prevalent conformation	binding affinity (kcal/mol)	–6.32	–5.03
	inhibition constant (*K*_i_, μM)	23.15	206.62
	rank (cluster/total)	6/46	6/54
	number of poses per cluster	109	123

a Docking simulations were performed using the AutoDock-based Lamarckian genetic algorithm (LGA) with 500 docking runs. The grid map was centered around the active site residues of trypanothione reductase (TR) in both oxidized (TR^ox^, PDB: 2JK6) and reduced (TR^red^, PDB: 4ADW) states.

TR is a homodimeric enzyme that features important binding sites that are important targets for identifying potential inhibitors. In addition to the catalytic triad (CYS52, CYS57, and HIS461) and substrate site (TYR110, LYS240, THR463, SER464, GLU18, GLU466, and GLU467), other sites, such as the Z-site (PHE396, PRO398, and LEU399) and hydrophobic wall (LEU17, TRP21, and MET113), are described as relevant attachment points.^31–34^ With a few exceptions, both ligands exhibited strong interactions with the catalytic sites of the *L. infantum* TR in both oxidation states. The same finding was noted for other local interactions. Both ligands, silibinin A and silibinin B, interacted with important residues at these sites. To achieve lower-energy and more prevalent conformations, silibinin A interacts with all residues of the catalytic site, all Z-site residues, and LEU17 of the hydrophobic wall in the oxidized TR (Figure 6a, Table 3). In the reduced form (reduced TR), silibinin A forms one hydrogen bond with CYS52 and one noncovalent interaction with HIS461. In addition, this structure interacts with all Z-site and hydrophobic wall residues (Figure 6b).

**Figure 6:**
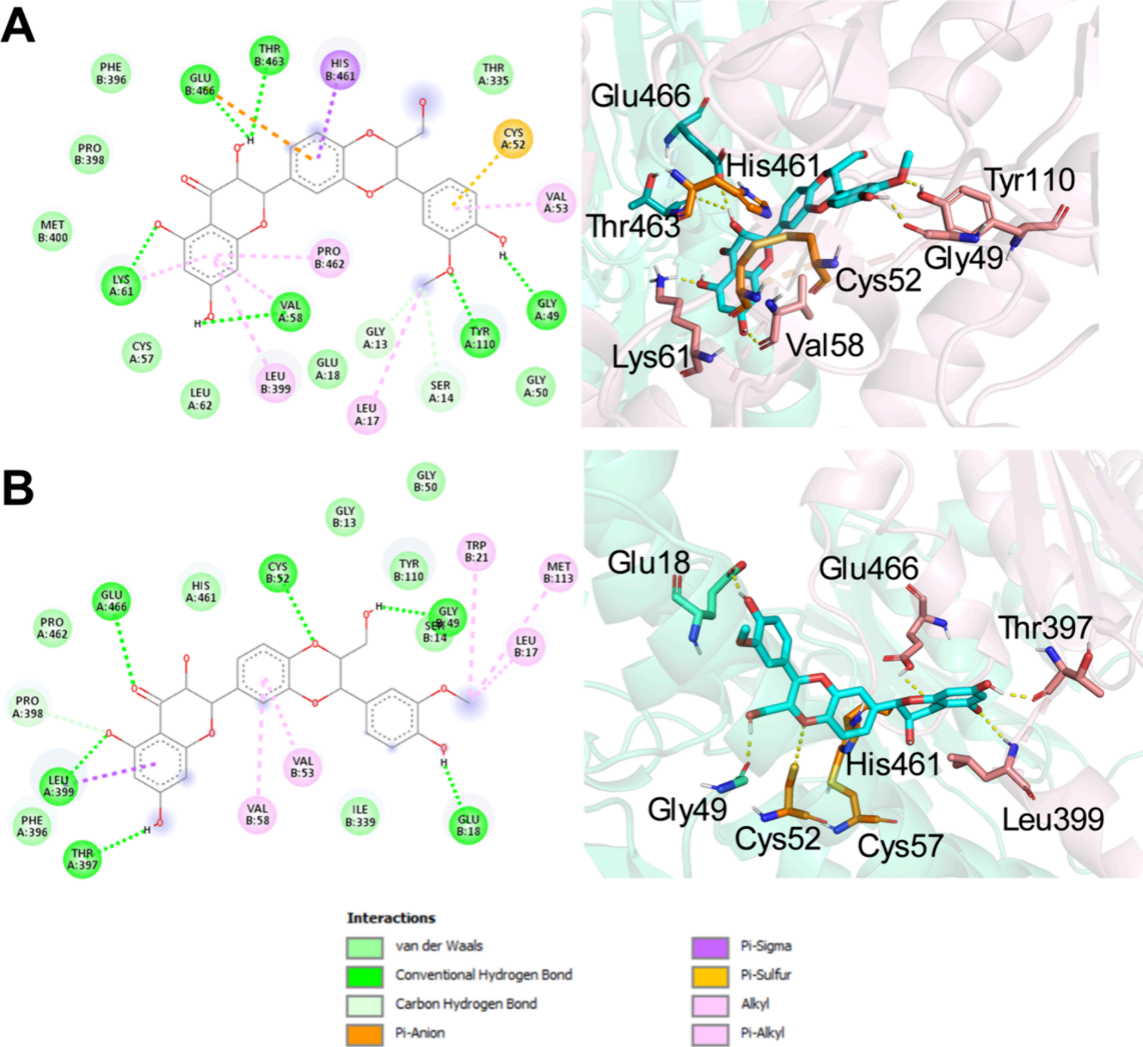
Conformational and molecular docking analyses of diastereoisomer A to the oxidized and reduced forms of trypanothione reductase (TR). Interactions between diastereoisomer A (cyan sticks) and oxidized (A) and reduced (B) TR (cartoon representation). The catalytic triad residues (CYS52, CYS57, and HIS461) are shown as orange sticks. Yellow dashes represent hydrogen bonding interactions. (A) 2D and 3D representations of diastereoisomer A bound to oxidized TR (2JK6) in its lowest energy and most prevalent conformation. (B) 2D and 3D representations of diastereoisomer A bound to reduced TR (4ADW) in its lowest energy conformation.

Additionally, silibinin B exhibited important interactions with both the oxidized and reduced forms of *L. infantum* TR. In the lower-energy conformation, silibinin B interacts with CYS52 and HIS461 from the catalytic site, forming four hydrogen bonds with different substrate residues, all Z-site residues and LEU17 from the hydrophobic wall (Figure 7a). Apart from these interactions, silibinin B in a lower-energy cluster of the reduced form exhibited two hydrogen bonds with HIS461 and all Z-site residues. On the other hand, one unfavorable donor–donor (H–H) interaction with the substrate residue GLU466 was observed (Figure 7b). These results strongly suggest that silibinin could be a competitive inhibitor of *L. infantum* TR, which may explain the correlation between ROS accumulation and the inhibition of *L. infantum* in promastigotes (Figure 4) that ultimately triggers both promastigote and amastigote death.

**Figure 7:**
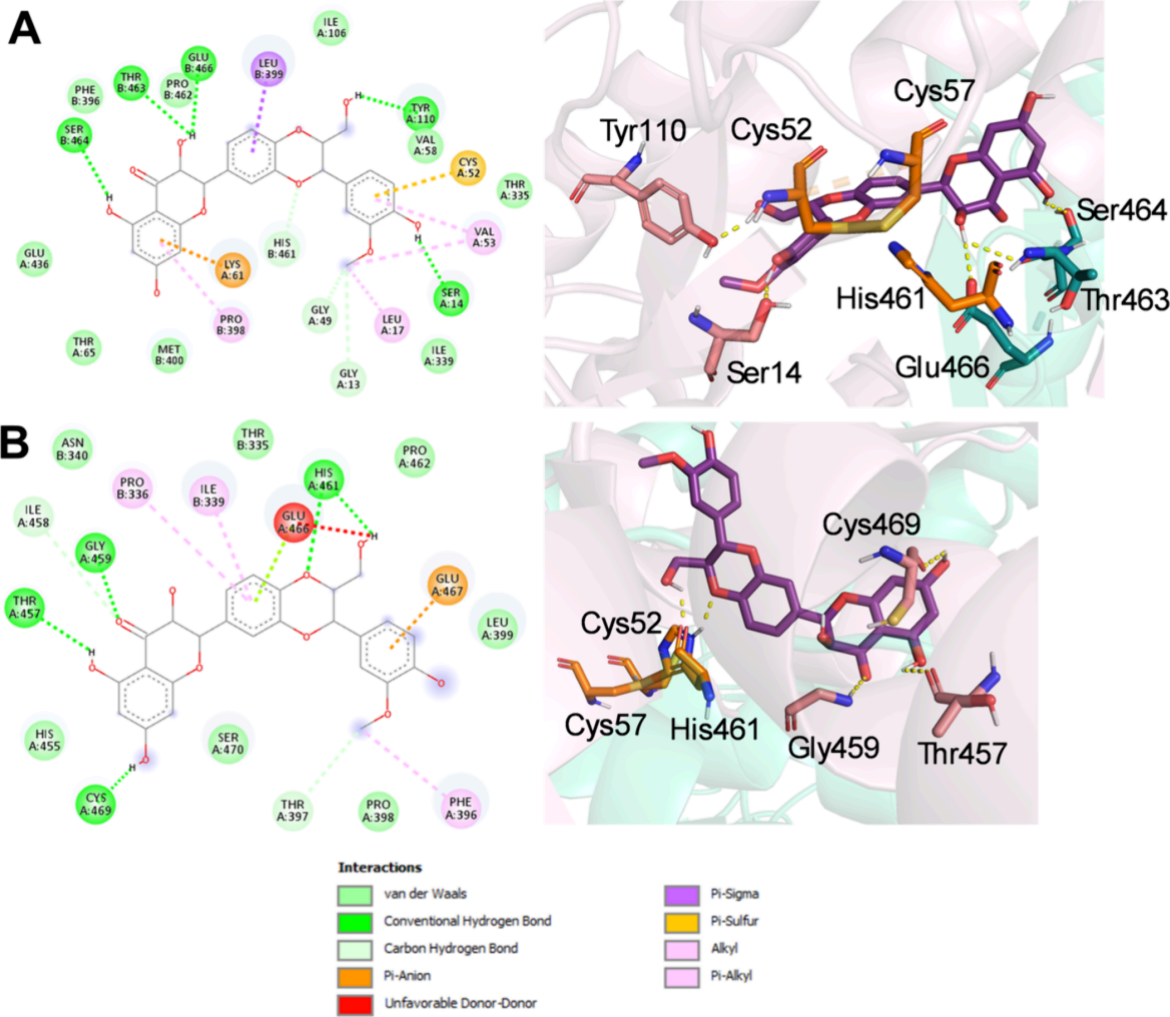
Conformational and molecular docking analyses of diastereoisomer B to the oxidized and reduced forms of trypanothione reductase (TR). Interactions between diastereoisomer B (purple sticks) and oxidized (A) and reduced (B) TR (cartoon representation). The catalytic triad residues (CYS52, CYS57, and HIS461) are shown as orange sticks. Yellow dashes indicate hydrogen bonding interactions. (A) 2D and 3D representations of diastereoisomer B bound to oxidized TR (2JK6) in its lowest energy conformation. (B) 2D and 3D representations of diastereoisomer B bound to reduced TR (4ADW) in its lowest energy conformation.

To provide experimental evidence supporting the *in silico* prediction that silibinin can interact with and inhibit TR, we conducted *in vitro* enzymatic assays to assess TR activity in the presence of this flavonoid at 500 μM. These results, as shown in Figure 8, demonstrate that silibinin inhibits TR activity, reaching 85% inhibition.

**Figure 8:**
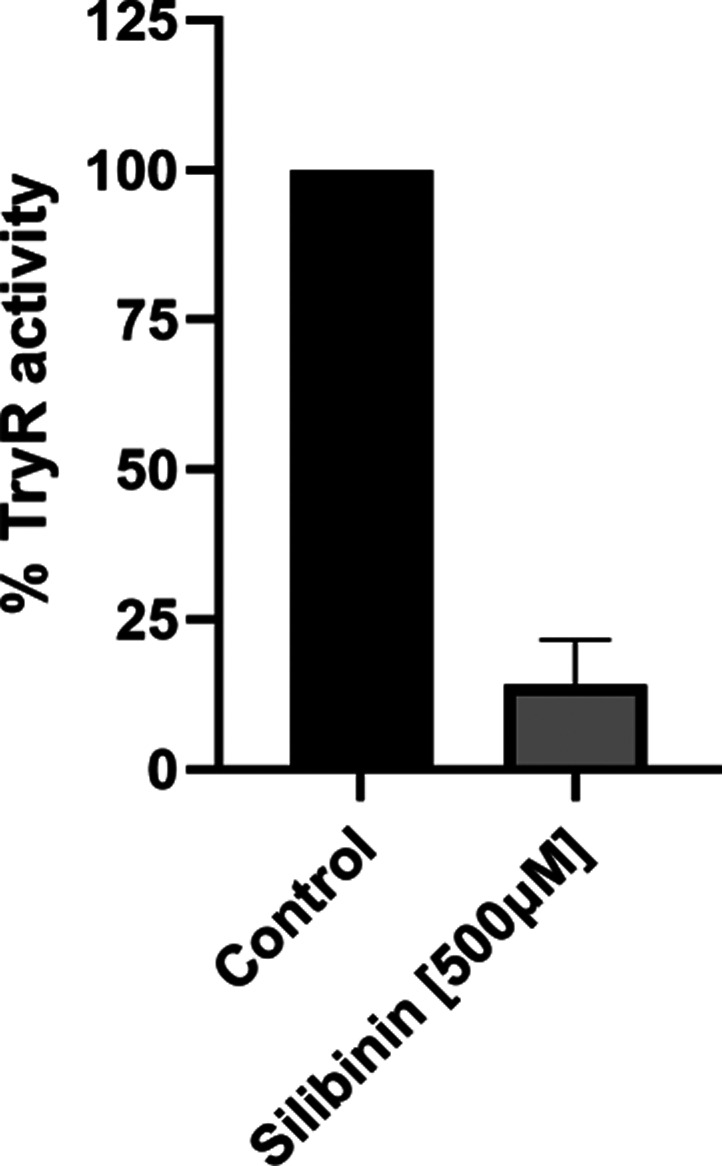
Silibinin inhibited *L. infantum* trypanothione reductase activity. *L. infantum* TryR activity was assayed at 25 °C and pH 7.5 in the presence of 500 μM silibinin in triplicate. Control experiments were carried out in the absence of the inhibitor. Values represent the means ± standard errors of three experimental replicates performed in triplicate.

The inhibition profile observed in this study aligns closely with findings in the literature, highlighting TR as a crucial target for antileishmanial compounds. Evidence has shown a significant correlation between ROS accumulation and TR inhibition induced by cyclobenzaprine in *L. infantum* promastigotes, where increased ROS levels were directly linked to reduced parasite viability.^35^ The ability of natural products to inhibit TR has also been well established. *Corchorus capsularis* L. leaf-derived β-sitosterol has been shown to exert antipromastigote effects against *L. donovani* through mechanisms involving the generation of ROS and competitive inhibition of TR, ultimately triggering apoptotic pathways.^36^ Similarly, the flavonoid epigallocatechin-3-gallate was shown to inhibit *L. infantum* promastigote proliferation, significantly reduce the infection index in macrophage cultures, and lower parasite burdens in murine models of visceral leishmaniasis. These effects were strongly associated with ROS production, biochemical alterations, and TR inhibition.^20,37^ Consistent with these findings, the present data suggest that silibinin exerts antileishmanial activity against *L. infantum* via competitive TR inhibition and enhanced ROS accumulation, which together appear to disrupt redox homeostasis and promote parasite death. These data reinforce the potential of TR as a therapeutic target for the development of novel leishmaniasis treatments.

## Conclusions

This study demonstrated the potential of silibinin as a promising candidate for the treatment of leishmaniasis. The leishmanicidal effects of silibinin against *L. infantum* promastigotes and intracellular amastigotes appear to be mediated by mechanisms involving trypanothione reductase (TR) inhibition and parasite redox homeostasis disruption, ultimately leading to cell death. In addition to its *in vitro* efficacy, theoretical predictions of the oral bioavailability of silibinin further support its potential for development as a chemotherapeutic agent. These results encouraged us to explore the efficacy of silibinin *in vivo* and reinforce the significance of targeting TR for leishmaniasis treatment. In further studies, the pharmacokinetics of silibinin should be optimized, and the effectiveness of this compound against different *Leishmania* spp. should be assessed to enhance the therapeutic arsenal for the treatment of this neglected tropical disease.

## Experimental Section

### Test Compound and Reagents

Silibinin (C_25_H_22_O_10_, molecular weight: 482.4 g/mol, purity 99.7% by HPLC; lot BCCD1066), Schneider’s *Drosophila* medium, fetal calf serum, and RPMI 1640 medium were obtained from Merck Sigma-Aldrich (St. Louis, Missouri, USA). Other reagents used in the study were purchased from Merck (São Paulo, Brazil). All of the solutions were prepared with deionized distilled water, which was obtained via a Milli-Q water purification system (Millipore Corp., Bedford, Massachusetts, USA). Throughout the experiments, only endotoxin-free sterile materials were utilized.

### Ethics Statement

This research was conducted in full compliance with the ethical guidelines set by the Brazilian National Council for the Control of Animal Experimentation (CONCEA). The experimental protocol was reviewed and approved by the Ethics Committee for Animal Experiments at Instituto Oswaldo Cruz (CEUA-IOC, License Number: L-11/2017).

### Parasites and Mice

The strain MHOM/MA/67/ITMAP263 of *L. infantum* was used in all of the experiments. Amastigotes were isolated from BALB/c mice and maintained as promastigotes in Schneider’s medium supplemented with 20% fetal bovine serum (v/v), 100 U/mL penicillin, and 100 μg/mL streptomycin at 26 °C. The parasites were passaged every 3 days to ensure maintenance. Female BALB/c mice (8–10 weeks old) were provided by the Instituto de Ciência e Tecnologia em Biomodelos (ICTB/FIOCRUZ). The animals were housed and bred at Instituto Oswaldo Cruz in compliance with the CONCEA guidelines for the care and use of laboratory animals.

### Promastigote Proliferation Assay

Promastigotes of *L. infantum* (1 × 10^6^ cells/mL) were incubated with various concentrations of silibinin (300−1000 μM) or a vehicle control (DMSO 0.2%) for 72 h. After incubation, the cell density was assessed using Alamar Blue (10% v/v), with absorbance measurements taken at 570 nm via a spectrophotometer. The initial growth curve was generated from a starting concentration of 1 × 10^6^ cells/mL. The IC_50_ (50% inhibitory concentration) was determined via logarithmic regression analysis via GraphPad Prism 6 (GraphPad Software, La Jolla, California, USA). All of the assays were performed in triplicate across three independent experiments.

### Leishmania-Macrophage Intracellular Assay

Peritoneal macrophages were harvested from BALB/c mice, cultured in RPMI 1640 medium, and plated at a density of 2 × 10^6^ cells/mL (0.4 mL per well) on Lab-Tek eight-chamber slides (Thermo Scientific, Waltham, Massachusetts, USA). The slides were incubated at 37 °C with 5% CO_2_ for 1 h. Stationary-phase *L. infantum* promastigotes were washed with PBS, counted using a Neubauer chamber, and added to the macrophages at a multiplicity of infection (MOI) of 5:1. The cultures were incubated for 4 h at 37 °C in 5% CO_2_. Excess parasites were removed by washing with RPMI 1640 medium. After an 18 h infection period, the macrophages were treated with various concentrations of silibinin (0.5−7.5 μM) in RPMI 1640 medium supplemented with 2% heat-inactivated horse serum for 72 h. Macrophage infections were quantified via light microscopy after staining with InStat Prov (Newprov, Curitiba, Brazil), with at least 200 cells counted per coverslip in duplicate. The infection index was calculated as the percentage of infected macrophages multiplied by the number of amastigotes per macrophage and expressed as percentage. EC_50_ was determined via logarithmic regression using GraphPad Prism 6. In the control group (absence of silibinin), 0.2% DMSO (vehicle) was added to the RPMI 1640 medium. The 100% value refers to 307.3 ± 51.5.

### Macrophage Cytotoxicity Assay

Peritoneal macrophages were collected from BALB/c mice and plated on 96-well tissue culture plates in RPMI 1640 medium at a density of 2 × 10^6^ macrophages/mL (0.2 mL/well) for 1 h at 37 °C in an atmosphere of 5% CO_2_. Nonadherent cells were removed by washing with an RPMI 1640 medium. The adherent macrophages were then incubated with the indicated concentrations of silibinin (0.98–1000 μM) for 72 h. The medium was discarded, and the macrophages were washed with RPMI 1640 and incubated with Alamar Blue (10% v/v) for 12 h at 37 °C in an atmosphere of 5% CO_2_. The fluorescence was measured at λ excitation 560 nm and λ emission 590 nm using a fluorimeter, and the CC_50_ value was determined by logarithmic regression analysis using GraphPad Prism 9. The selectivity index was determined as the macrophage CC_50_/intracellular amastigote EC_50_. In the control group (absence of silibinin), 0.2% DMSO (vehicle) was added to the RPMI 1640 medium.

### *In Silico* Parameters and ADMET Properties of Silibinin

The physicochemical parameters of silibinin were available to study its ADMET properties and oral bioavailability. The SMILES string of silibinin, COC1=C­(C=CC­(=C1)­[C@@H]­2­[C@H]­(OC3=C­(O2)­C=C­(C=C3)­[C@@H]­4­[C@H]­(C­(=O)­C5=C­(C=C­(C=C5O4)­O)­O)­O)­CO)­O, was obtained from PubChem (CID 31553) and input to the pkCSM platform^14^ to evaluate the ADMET parameters and physicochemical characteristics for Lipinski’s Rule of Five.^15,16^ To corroborate these analyses, Bioavailability Radar on the SwissADME platform^38^ was used with the exact SMILES string as the input.

### ROS Measurement

Intracellular ROS levels were measured in *L. infantum* promastigotes treated with different concentrations of silibinin (300, 350, 400, and 450 μM) for 72 h. Cells were harvested, resuspended in Hanks' balanced salt solution (HBSS), adjusted to a concentration of 2 × 10^6^/mL, and incubated with the probe 2′,7′-dichlorodihydrofluorescein diacetate (H_2_DCFDA; 20 μM; Invitrogen Molecular Probes, Leiden, The Netherlands) for 20 min at 26 °C. ROS levels were measured spectrofluorometrically at λ excitation 485 nm and λ emission 530 nm; 1 mM H_2_O_2_ was used as a positive control. The linear correlation with the antipromastigote effect and the corresponding Pearson correlation coefficient were evaluated using GraphPad Prism 9 software.

### Docking Studies

The crystal structures of the oxidized and reduced forms of *L. infantum* TR (PDB 2JK6 and 4ADW, respectively)^25^ were downloaded from the RCSB Protein Data Bank (www.rcsb.org). The atomic partial charges were calculated with the PDB 2PQR Server (www.nbcr-222.ucsd.edu) using the AMBER force field, and the protonation states at pH 7.4 were determined using PROKA. All proteins were converted to PDBQT format using AutoDock Tools. The structures of the silibinin diastereoisomers silibinins A and B were prepared using AutoDock Tools. A cubic grid with sides of 80 Å and a spacing of 0.375 Å was constructed centered on the active site residues (*x*, *y*, *z* coordinates: 23.3, 51.9, −15.6) of both the oxidized and reduced TR structures^18^ using AutoDock Tools. The Lamarckian genetic algorithm (LGA) was applied for 500 simulations. Ligand binding position and interaction analyses were performed using PyMOL software, and the 2D interactions were visualized with BIOVIA Discovery Studio 2020 (Dassault Systems BIOVIA, Discovery Studio Modeling Environment, Release 2017, San Diego: Dassault Systems, 2016).

### TR Activity Assay

This assay was performed in 96-well plates on the basis of the generation of reduced trypanothione [T­(SH)­2] by TR, which is coupled with the chemical oxidant 5,5′-dithiobis­(2-nitrobenzoic acid) (DTNB, Ellman’s reagent).^39,40^ Each reaction mixture consisted of 40 mM Tris–HEPES (pH 7.05), 1 mM EDTA, 30 μM NADP+, 1 μM oxidized trypanothione (TS2), 0.1 mM DTNB, 0.1 mM NADPH, and 500 μM of silibinin. The reaction was started by the addition of the soluble fraction of *L. infantum* (0.1 mg/mL). Then, the rate of TNB formation at 25 °C was monitored at 410 nm for 60 min using a spectrophotometer. To obtain the soluble fraction, 3-day cultures of *Leishmania infantum* promastigotes were centrifuged at 1000*g* for 10 min. Following centrifugation, the supernatant was discarded, and the pellet was resuspended in a protease inhibitor solution consisting of 40 mM Tris–Hepes buffer (pH 7.5), 1 mM EDTA (pH 8), 250 mM sucrose, 10 mM KCl, 1 mM PMSF, and 10 μM E-64. The suspension was subsequently subjected to thermal shock by immersion in liquid nitrogen, followed by further centrifugation at 17,500*g*. The supernatant obtained after this second centrifugation was designated as the soluble fraction. The protein concentration in the soluble fraction was quantified using a NanoDrop Lite Plus spectrophotometer.

### Statistical Analysis

Each experiment was conducted three times independently. The data were analyzed using the Mann–Whitney test or one-way ANOVA followed by Tukey’s post hoc test using GraphPad Prism 6. Differences with a *p* value of ≤ 0.05 were considered statistically significant. The data are presented as means ± standard errors.
